# Plant Polyphenol Gossypol Induced Cell Death and Its Association with Gene Expression in Mouse Macrophages

**DOI:** 10.3390/biom13040624

**Published:** 2023-03-30

**Authors:** Heping Cao, Kandan Sethumadhavan

**Affiliations:** United States Department of Agriculture, Agricultural Research Service, Southern Regional Research Center, New Orleans, LA 70124, USA

**Keywords:** cytokine, gene expression, glucose transport, gossypol, inflammation, insulin signaling, plant polyphenol, toxicity, tristetraprolin

## Abstract

Gossypol is a complex plant polyphenol reported to be cytotoxic and anti-inflammatory, but little is known about its effect on gene expression in macrophages. The objective of this study was to explore gossypol’s toxicity and its effect on gene expression involved in the inflammatory response, glucose transport and insulin signaling pathways in mouse macrophages. Mouse RAW264.7 macrophages were treated with multiple concentrations of gossypol for 2–24 h. Gossypol toxicity was estimated by MTT assay and soluble protein content. qPCR analyzed the expression of anti-inflammatory tristetraprolin family (TTP/ZFP36), proinflammatory cytokine, glucose transporter (GLUT) and insulin signaling genes. Cell viability was greatly reduced by gossypol, accompanied with a dramatic reduction in soluble protein content in the cells. Gossypol treatment resulted in an increase in TTP mRNA level by 6–20-fold and increased ZFP36L1, ZFP36L2 and ZFP36L3 mRNA levels by 26–69-fold. Gossypol increased proinflammatory cytokine TNF, COX2, GM-CSF, INFγ and IL12b mRNA levels up to 39–458-fold. Gossypol treatment upregulated mRNA levels of GLUT1, GLUT3 and GLUT4 genes as well as INSR, AKT1, PIK3R1 and LEPR, but not APP genes. This study demonstrated that gossypol induced macrophage death and reduced soluble protein content, which was accompanied with the massive stimulation of anti-inflammatory TTP family and proinflammatory cytokine gene expression, as well as the elevation of gene expression involved in glucose transport and the insulin signaling pathway in mouse macrophages.

## 1. Introduction

Plant bioactive extracts have been used for disease prevention and treatment since ancient times. One group of the major bioactive compounds in plant extracts is plant polyphenols [1]. They are produced from the plant flavonoid biosynthetic pathway and used naturally for plant defenses against predators [2]. Plant polyphenols are present in most diets and are beneficial to human health [3,4,5,6]. 

**Figure 1:**
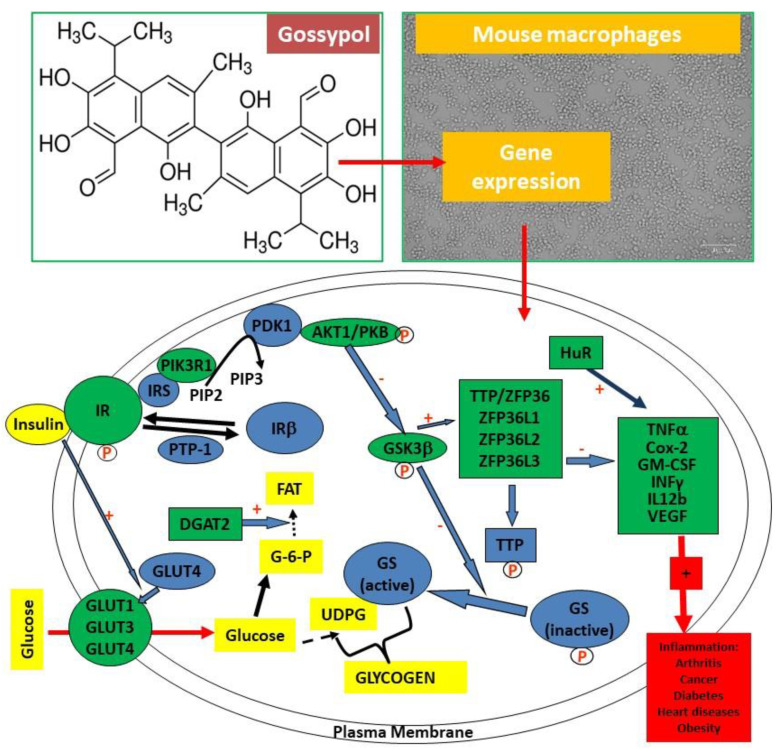
Gossypol, mouse RAW264.7 macrophages and biomarkers analyzed by qPCR. (**Top left**) Gossypol is a plant polyphenol with six -OH groups and six -CH_3_ groups. (**Top right**) Mouse RAW264.7 macrophages used in the study. (**Bottom**) Gene targets analyzed by qPCR in this study (highlighted in green color).

Gossypol is a complex plant polyphenol with six OH groups and six CH_3_ groups in its molecule, found in the small intercellular pigment glands in cotton plants, especially in the glanded cottonseed (Figure 1) [7]. Long-term consumption of cottonseed oil with a high concentration of gossypol contributes to its toxicity, resulting in male infertility [8]. The high concentration of gossypol in cottonseed meal (protein products) also limits its uses [9,10]. Therefore, gossypol is traditionally regarded as unsafe for most animals and human consumption. However, recent studies have demonstrated that gossypol and related compounds have anticancer activities, including against breast cancer [11], colon cancer [12], pancreatic cancer [13,14] and prostate cancer [15,16]. These discoveries suggest the potential nutritional and/or medical utilization of gossypol and related compounds.

It was reported that gossypol has anti-inflammatory activities in cultured macrophages [17,18]. However, this area of research has been poorly studied, and little is known about its inflammatory effects in mammalian cells. Recently, it was shown that tristetraprolin/zinc finger protein 36 (TTP/ZFP36) family proteins are anti-inflammatory [19,20]. These RNA-binding proteins regulate gene expression at the posttranscriptional level by destabilizing proinflammatory cytokine mRNA molecules in mammalian cells. TTP family proteins bind to AU-rich elements (AREs) in proinflammatory cytokine mRNAs and destabilize those molecules [21,22]. TTP knockout mice accumulate proinflammatory cytokines and develop a severe systemic inflammatory syndrome, including arthritis, autoimmunity and myeloid hyperplasia [23,24]. Upregulation of TTP decreases inflammatory responses in macrophages [25]. These data suggest that TTP is an anti-inflammatory protein and arthritis suppressor. Chemicals that can increase TTP gene expression may have therapeutic value for the prevention and/or treatment of inflammation-related diseases. However, no studies have been performed to relate gossypol to TTP-mediated anti-inflammatory effects.

Plant polyphenols have been shown to regulate mammalian gene expression in numerous studies. For example, green tea polyphenols regulate gene expression in rats with metabolic syndrome caused by a high-fructose diet [26,27]. Cinnamon polyphenols regulate the expression of genes coding for proteins in the insulin signaling pathway, inflammatory responses and lipid metabolism [28,29,30,31]. However, little is known of whether gossypol regulates gene expression involved in glucose transport and insulin signaling.

The objective of this study was to explore the toxicity and molecular effects of gossypol on the expression of genes involved in the inflammatory response, glucose transport and insulin signaling pathways in mouse RAW264.7 macrophages, a well-characterized cell model for inflammatory research (Figure 1). The targets of gene expression analysis included anti-inflammatory TTP family genes (TTP/ZFP36, ZFP36L1, ZFP36L2 and ZFP36L3) [32,33] and proinflammatory cytokine genes, such as tumor necrosis factor (TNF/TNFα) [21], cyclooxygenase 2/prostaglandin-endoperoxide synthase 2 (COX2/PGES2) [34], granulocyte-macrophage colony stimulating factor (GM-CSF/CSF2) [35], interferon gamma (INFγ) [36] and interleukin 12 (IL12) [37] (Figure 1). Other targets included the glucose transporter family (GLUT1, GLUT2, GLUT3 and GLUT4), critically important for host immunity [38,39]; and some components in the insulin signaling pathway, including insulin receptor (INSR) and protein kinases AKT1, GSK3β and PIK3R1, which are shown to be important in insulin resistance in macrophages [26,40] (Figure 1). The results showed that gossypol (input) induced macrophage death, reduced protein content and stimulated the expression of genes coding for the anti-inflammatory TTP family, proinflammatory cytokine, GLUT family and insulin signaling pathway components in mouse RAW264.7 macrophages (output).

## 2. Materials and Methods

### 2.1. Cell Line, Chemicals and Reagents

Mouse RAW264.7 macrophages were from American Type Culture Collection (Manassas, VA, USA). Cell culture reagents were from Gibco BRL (Thermo Fisher, Waltham, MA, USA). Gossypol (G8761-100MG, (+/−)-gossypol from cotton seeds, ≥95% pure) and dimethylsulfoxide (DMSO) were from Sigma (St. Louis, MO, USA). TRIzol was from Thermo Fisher (Waltham, MA, USA). SuperScript II reverse transcriptase, oligo(dT)_12–18_ primer, random primers, dNTPs, DTT and RNaseOUT were from Life Technologies. CFX96 real-time system-C1000 Thermal Cycler, 1× iQ SYBR Green Supermix and qPCR assay accessories were from Bio-Rad (Hercules, CA, USA). PCR primers were designed using Primer Express software (Thermo Fisher) and synthesized by Biosearch Technologies (Petaluma, CA, USA) (Supplementary Table S1).

### 2.2. Cell Culture and Treatment

Mouse macrophages were maintained at 37 °C in a water jacket CO_2_ incubator with 5% CO_2_ in DMEM containing 4.5 mg/mL (25 mM) glucose supplemented with 10% (*v*:*v*) fetal bovine serum, 100 units/mL penicillin, 100 µg/mL streptomycin and 2 mM L-glutamine as described. The experiments were started with the same number of RAW macrophages (0.5 mL, 1 × 10^5^ cells/mL) subcultured in 24-well cell plate. Raw macrophages were treated with various concentrations of gossypol dissolved in 100% DMSO for different times, as detailed below. The control and all treatments contained 1% DMSO in the culture medium.

### 2.3. Cell Toxicity Assay

MTT-Based In Vitro Toxicology Assay Kit (TOX1-1KT, Sigma, St. Louis, MO, USA) was used to determine mouse macrophage toxicity as described previously [41]. MTT assay is based on the conversion of water-soluble MTT (thiazolyl blue tetrazolium bromide) to an insoluble formazan product by viable cells with active metabolism. Dead cells lose this ability, and therefore show no signal. The more metabolic activity in the sample, the higher the signal. The selection of gossypol concentration and duration of treatment was based on our previous study using human colon cancer cells [42,43]. Mouse macrophages were treated with up to 100 µg/mL of gossypol and incubated at 37 °C, 5% CO_2_ for 2–24 h. MTT assay reagent was added to the media and incubated at 37 °C, 5% CO_2_ for 2 h before adding MTT solubilization solution to each well and shaking at room temperature overnight. The color density in the wells was recorded by Epoch microplate spectrophotometer at A570 nm.

### 2.4. Protein Determination

Mouse macrophages were treated with gossypol (100 mg/mL) for 2–24 h. Cell extracts were prepared according to a previously described procedure [44]. Protein concentrations were determined with the Bradford method using the Bio-Rad reagent (Bio-Rad) [45].

### 2.5. RNA Extraction, cDNA Synthesis and Real-Time qPCR Analysis

The qPCR assays followed the MIQE guidelines: minimum information for publication of quantitative real-time PCR experiments [46]. The qPCR assays were described in detail previously [47]. Raw macrophages were treated with up to 100 µg/mL of gossypol for 2–24 h. RNAs were isolated from macrophages using TRIzol reagent. The cDNAs were synthesized from total RNAs essentially as described [48]. SYBR Green qPCR reaction mixtures and the thermal cycle conditions were identical to those described [48]. The ΔΔ*C_T_* method of relative quantification was used to determine the fold change in gene expression [49]. First, the cycle of threshold (*C_T_*) was obtained from 3–6 independent samples. Second, the first delta *C_T_* value (Δ*C_T_*) was obtained by subtracting the *C_T_* value of the internal reference control (mouse 60S ribosome protein 32, Rpl32) [50] from the *C_T_* value of the target mRNA (Δ*C_T_* = *C_T_*_Target_ − *C_T_*_ref_). Third, the second delta *C_T_* value (ΔΔ*C_T_*) was obtained by subtracting the Δ*C_T_* of the calibrator (1% DMSO control in the figures or Ttp in Table 3 from the Δ*C_T_* of the target mRNA (ΔΔ*C_T_* = Δ*C_T_*_Target_ − Δ*C_T_*_cal_). Finally, the fold change in expression was obtained using the equation 2^−ΔΔ^*^CT^*.

### 2.6. Statistics

The data represent the mean and standard deviation of 3–6 independent samples. They were analyzed using ANOVA with SigmaStat 3.1 software (Systat Software). Multiple comparisons among the treatments with various concentrations of gossypol in each treatment time were performed with Student–Newman–Keuls Method. “*” and “**” displayed in the Tables and Figures represent significant differences between the control and the treatment at *p* < 0.05 and *p* < 0.01, respectively.

## 3. Results

### 3.1. Gossypol Inhibited Mouse Macrophages Growth

**Figure 2:**
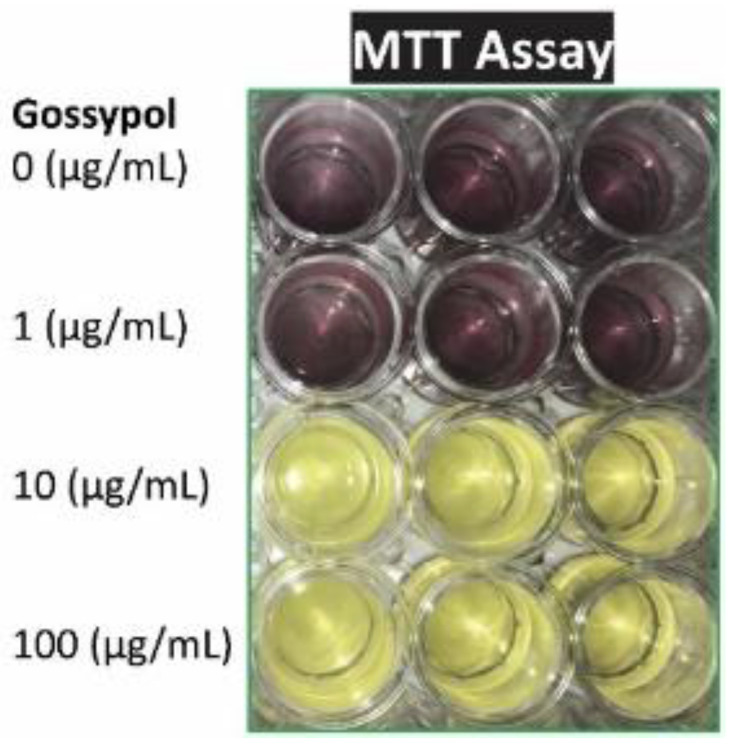
Gossypol effect on cell viability. Mouse RAW264.7 macrophages were treated with various concentrations of gossypol for 24 h (triplicate). MTT assay reagent was added to the media and incubated for 2 h before adding MTT solubilization solution.

Macrophage viability was measured by visualization and with MTT assay after cells were treated with the cytotoxic compound gossypol. Gossypol exhibited a significant inhibitory effect on mouse macrophage growth under higher concentration (10–100 µg/mL for 2 h) or longer treatment time (5–100 µg/mL for 24 h) (Figure 2 and Table 1). Gossypol treatment significantly reduced RAW macrophage viability to 20% of the control by 100 µg/mL for 2 h or less than 10% of the control by 5–100 µg/mL for 24 h (Table 1). The higher-concentration treatment resulted in lower A570 nm, meaning fewer viable/metabolic active cells under higher-concentration treatment (Table 1).

### 3.2. Gossypol Reduced Soluble Protein Content in Mouse Macrophages

Another indication for gossypol toxicity on macrophages was its effect on soluble protein content in mouse macrophages, since it is generally accepted that the total soluble protein content in the cell reflects the overall health status of cellular metabolism. The soluble protein content was dramatically reduced by 16, 17, 38 and 97% in cells treated with gossypol for 2, 4, 8 and 24 h, respectively, although the protein content recovered in the pellet was slightly higher in gossypol-treated macrophages (Table 2). Gossypol treatment resulted in a reduction in total protein by 17 and 76% in macrophages treated for 8 and 24 h, respectively (Table 2). Both MTT assay and protein determination indicated that gossypol was toxic to mouse RAW264.7 macrophages.

### 3.3. Relative Expression Levels of Selected Genes in Mouse Macrophages

For better comparison studies, we first used quantitative real-time PCR to evaluate the relative mRNA levels of the selected genes in mouse RAW264.7 cells treated with 1% DMSO control for 24 h using RPL32 as the internal control and TTP/ZFP36 as the calibrator (Table 3). TTP/ZFP36 family genes, including ZFP36L1, ZFP36L2 and ZFP36L3, were expressed at approximately 0.40-, 1.11- and 0.05-fold of TTP, respectively, in the DMSO-treated macrophages (Table 3). The mRNA levels of proinflammatory cytokine genes, including TNF, COX2, GM-CSF, IFN*γ* and IL12b, were 0.05-, 0.02-, 0.14-, 0.13- and 0.10-fold of TTP, respectively (Table 3). Although macrophages are not model cells for glucose transport and insulin signaling research, significant amounts of mRNAs coding for GLUT family and insulin signaling components were detected in the mouse macrophages (Table 3). GLUT1, GLUT2, GLUT3 and GLUT4 were approximately 0.14-, 0-, 0.29- and 0.001-fold of TTP, respectively. INSR, PIK3R1 and LEPR mRNAs were 0.32-, 0.37- and 0.08-fold of TTP, and those of AKT1 and APP were 2.16- and 4.76-fold of TTP, respectively (Table 3).

### 3.4. Gossypol Increased TTP Family Gene Expression in Mouse Macrophages

**Figure 3:**
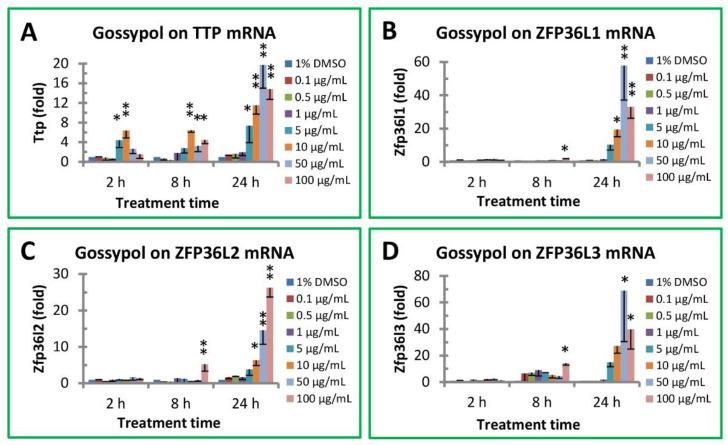
Effect of gossypol on TTP family gene expression. (**A**) TTP/ZFP36 mRNA, (**B**) ZFP36L1 mRNA, (**C**) ZFP36L2 mRNA, (**D**) ZFP36L3 mRNA. RAW264.7 macrophages were treated with gossypol (0–100 µg/mL) for 2–24 h. The SYBR Green qPCR reaction mixtures contained 5 ng of RNA-equivalent cDNAs from each sample and 200 nM of each primer. The 2^−ΔΔ^*^CT^* method of relative quantification was used to determine the fold change in expression using RPL32 mRNA as the reference mRNA. The data represent the mean and standard deviation of three independent samples. “*”and “**” displayed in the Figure represent significant differences between the control and the treatment at *p* < 0.05 and *p* < 0.01, respectively.

Mouse TTP family genes have four members coding for anti-inflammatory TTP and its three TTP homologues: ZFP36L1, ZFP36L2 and ZFP36L3. Gossypol significantly increased TTP mRNA levels in mouse macrophages (Figure 3A). TTP mRNA levels were increased more than 6-fold by gossypol after 2 h treatment, and the effect was sustained after 8 h treatment (>6-fold) and even stronger stimulation after 24 h treatment (about 20-fold) (Figure 3A). Gossypol increased ZFP36L1 mRNA levels approximately 2-fold under 2 and 8 h treatment but significantly increased its mRNA levels up to 58-fold under 24 h treatment (increasing 10-, 19-, 58- and 33-fold by 5, 10, 50 and 100 µg/mL of gossypol treatment, respectively) (Figure 3B). ZFP36L2 mRNA levels were also significantly increased by gossypol treatment under longer times or higher concentrations (increasing 4-, 6-, 15- and 26-fold by 5, 10, 50 and 100 µg/mL of gossypol treatment, respectively) (Figure 3C). The stimulatory effect of gossypol on ZFP36L3 gene expression was stronger than the other TTP family members under longer treatment times (Figure 3D). ZFP36L3 mRNA levels were not affected by gossypol after 2 h treatment but modestly increased for higher concentrations of gossypol under 8 h treatment (14-fold increase by 100 µg/mL of gossypol) and increased much more in 24 h treatment (15–69-fold by 5–50 µg/mL of gossypol) (Figure 3D).

### 3.5. Gossypol Increased Proinflammatory Cytokine Gene Expression in Mouse Macrophages

**Figure 4:**
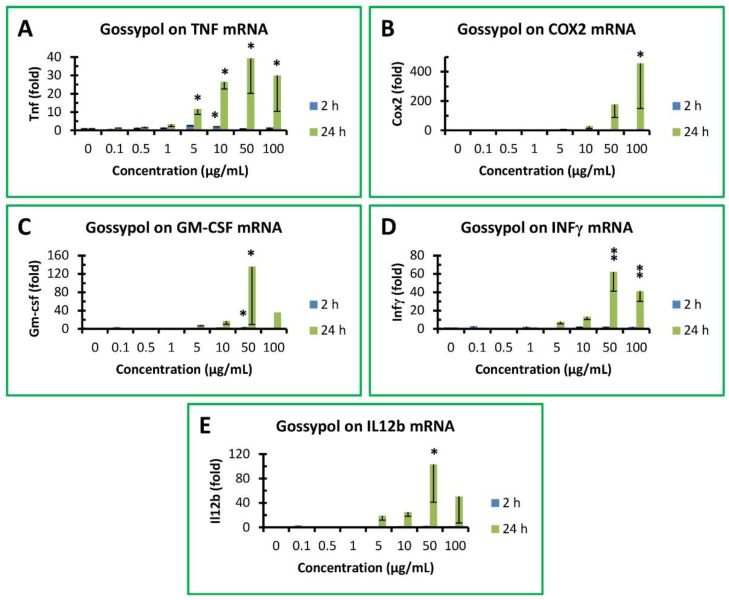
Effect of gossypol on proinflammatory cytokine gene expression. (**A**) TNF mRNA, (**B**) COX2 mRNA, (**C**) GM-CSF mRNA, (**D**) IFNγ mRNA, (**E**) IL12b mRNA. RAW264.7 macrophages were treated with gossypol (0–100 µg/mL) for 2–24 h. The data represent the mean and standard deviation of three independent samples. “*” and “**” displayed in the Figure represent significant differences between the control and the treatment at *p* < 0.05 and *p* < 0.01, respectively.

TTP is a mRNA-destabilizing factor for a number of proinflammatory cytokines, such as TNF, COX2, GM-CSF, INFγ and IL12b [32]. Therefore, expression of these proinflammatory cytokine genes was investigated in RAW264.7 macrophages after being treated with gossypol. Gossypol exhibited a less than 3-fold increase in TNF mRNA levels in RAW cells treated for 2 h, but increased its level to 12-, 27-, 39- and 30-fold after 24 h treatment with 5, 10, 50 and 100 µg/mL gossypol, respectively (Figure 4A). TNF mRNA levels were slightly but significantly increased by 5 and 10 µg/mL gossypol treatment for 2 h. However, TNF mRNA levels were dramatically increased by 10, 50 and 100 µg/mL gossypol treatment for 24 h (Figure 4A). Gossypol exhibited a much higher induction of COX2 gene expression to 9-, 30-, 177- and 458-fold after 24 h treatment with 5, 10, 50 and 100 µg/mL gossypol, respectively, although its effect on COX2 was less than 8-fold without statistical significance after 2 h treatment (Figure 4B). COX2 mRNA levels were significantly increased by 100 µg/mL gossypol treatment for 24 h (Figure 4B). Similarly, gossypol stimulation on GM-CSF mRNA levels was less than 5-fold under 2 h treatment but increased to 9-, 18-, 136- and 36-fold after 24 h treatment with 5, 10, 50 and 100 µg/mL gossypol, respectively (Figure 4C). GM-CSF mRNA levels were significantly increased by 50 µg/mL gossypol treatment for 2 or 24 h (Figure 4C). INFγ mRNA levels were increased to 8-, 13-, 62- and 41-fold after 24 h treatment with 5, 10, 50 and 100 µg/mL gossypol, respectively, but less than 4-fold under 2 h treatment without statistical significance (Figure 4D). INFγ mRNA levels were significantly increased by 50 and 100 µg/mL gossypol treatment for 24 h (Figure 4D). IL12b gene expression was less than 4-fold after 2 h treatment without statistical significance but increased to 19-, 25-, 103- and 51-fold after 24 h treatment with 5, 10, 50 and 100 µg/mL gossypol, respectively (Figure 4E). IL12b mRNA levels were significantly increased by 50 µg/mL gossypol treatment for 24 h (Figure 4E).

### 3.6. Gossypol Increased GLUT Family Gene Expression in Mouse Macrophages

**Figure 5:**
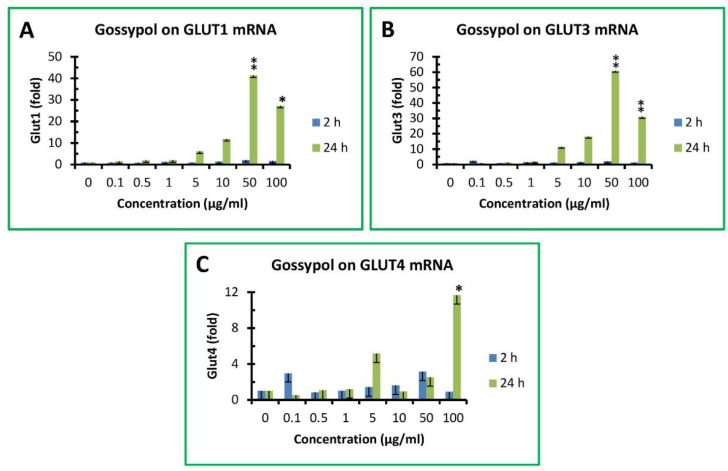
Effect of gossypol on glucose transporter gene expression. (**A**) GLUT1 mRNA, (**B**) GLUT3 mRNA, (**C**) GLUT4 mRNA. RAW264.7 macrophages were treated with gossypol (0–100 µg/mL) for 2 and 24 h. The data represent the mean and standard deviation of three independent samples. “*” and “**” displayed in the Figure represent significant differences between the control and the treatment at *p* < 0.05 and *p* < 0.01, respectively.

Glucose is a major metabolic substrate that is critically important for host immunity [38,39]. Glucose uptake in mammalian cells is facilitated by glucose transporter (GLUT) family proteins [51]. Gossypol slightly increased GLUT1 mRNA levels after 2 h treatment by approximately 2–3-fold of the control under 50 and 100 µg/mL treatment, respectively (Figure 5A). After 24 h treatment, GLUT1 mRNA levels in RAW cells were dramatically increased by 6-, 12-, 41- and 27-fold of the control under 30, 40, 50 and 100 µg/mL treatment, respectively (Figure 5A). Gossypol had a minor effect on GLUT3 mRNA levels under 2 h treatment (less than 2-fold of the control) but exhibited a significant effect by 12-, 18-, 61- and 31-fold of the control after 24 h treatment under 30, 40, 50 and 100 µg/mL treatment, respectively (Figure 5B). The effect of gossypol on GLUT4 mRNA levels was less than 3-fold of the control under 2 h treatment and increased to only 3- and 12-fold of the control after 24 h treatment under 50 and 100 µg/mL treatment, respectively (Figure 5C). GLUT2 was undetectable in its basal stage or under gossypol treatment in mouse macrophages (data not shown).

### 3.7. Gossypol Increased Insulin Signaling Pathway Gene Expression in Mouse Macrophages

**Figure 6:**
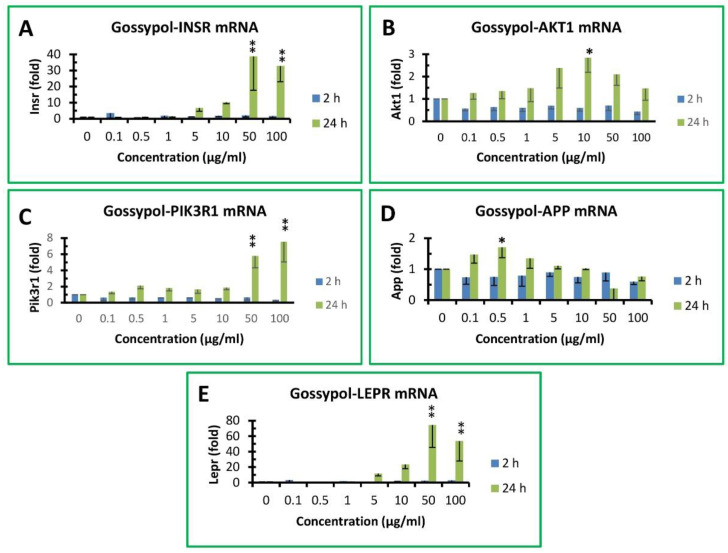
Effect of gossypol on insulin signaling pathway and other gene expression. (**A**) INSR mRNA, (**B**) AKT1 mRNA, (**C**) PIK3R1 mRNA, (**D**) APP mRNA, (**E**) LEPR mRNA. RAW264.7 macrophages were treated with gossypol (0–100 µg/mL) for 2 and 24 h. The data represent the mean and standard deviation of three independent samples. “*” and “**” displayed in the Figure represent significant differences between the control and the treatment at *p* < 0.05 and *p* < 0.01, respectively.

It was shown recently that macrophages express insulin receptors whose downstream signaling networks share a number of knots, allowing insulin to enhance or attenuate both proinflammatory and anti-inflammatory macrophage responses [40]. A few targets were therefore selected to determine if gossypol exhibited any effect on the expression of components involved in insulin signaling pathway, including INSR, AKT1, GSK3β and PIK3R1 (Figure 6). The effect of gossypol on INSR mRNA levels showed minimal effect after 2 h treatment but increased significantly to up to 39- and 32-fold of the control under 50 and 100 µg/mL treatment, respectively (Figure 6A). Gossypol did not appear to have a significant effect on AKT1 mRNA levels under 2 h treatment and increased to less than 3-fold of the control after 24 h treatment under various treatments (Figure 6B). The effect of gossypol on PIK3R1 mRNA levels was 6- and 8-fold of the control after 24 h treatment under 50 and 100 µg/mL treatment, respectively (Figure 6C). GSK3β mRNA levels were too low to be detected in macrophages under DMSO control or various gossypol treatment (data not shown).

### 3.8. Gossypol Effect on APP and LEPR Gene Expression in Mouse Macrophages

Since gossypol increased so many genes’ expression in mouse macrophages, we analyzed two unrelated genes for comparison, including APP and LEPR (Figure 6). APP gene expression was unresponsive to gossypol treatment under various concentrations for 2 or 24 with its mRNA levels less than 2-fold of the control (Figure 6D). On the other hand, gossypol’s effect on LEPR gene response was less than 3-fold of the control after 2 h treatment but significantly increased up to 12-, 23-, 74- and 54-fold of the control under 5, 10, 50 and 100 µg/mL treatment, respectively (Figure 6E).

## 4. Discussion

In this study, we examined the effects of gossypol from cottonseed on the cell viability, protein accumulation and mRNA levels of anti-inflammatory TTP family genes (coding for mRNA-destabilizing proteins) and some TTP-mediated proinflammatory cytokine genes in mouse macrophages. We also evaluated gossypol effects on the expression of genes coding for glucose transporters and insulin signaling pathway components in mouse macrophages. Our results showed that gossypol inhibited cell growth and reduced soluble protein content, which was associated with elevated levels of mRNAs coding for proteins involved in the inflammatory response, glucose transport and insulin signaling pathways, as highlighted with the “green” color of the diagram (Figure 1).

The results from this study and several previous studies suggest that gossypol is a strong stimulator of gene expression in mouse macrophages for the following reasons: (1) gossypol increases mRNA-destabilizing anti-inflammatory TTP family gene expression (TTP/ZFP36L1, ZFP36L2, ZFP36L2 and ZFP36L3) (this study); (2) gossypol increases proinflammatory cytokine gene expression (this study); (3) gossypol increases mRNA-stabilizing human antigen R (HuR) gene expression [52]; (4) gossypol increases vascular endothelial growth factor (VEGF) gene expression [53]; (5) gossypol increases glucose transporter gene expression (this study); (6) gossypol increases insulin signaling pathway gene expression (INSR, AKT1 and PIK3R1) (this study); (7) gossypol increases diacylglycerol acyltransferase (DGAT) gene expression [48]. However, since gossypol induced cell death at the same time, it is still to be determined if the gene expression effect was due to the direct or indirect effect of gossypol in the cells.

We consistently observed that gossypol caused cell death and dramatically reduced soluble protein content in the mouse macrophages. Gossypol (5–100 µg/mL) decreased mitochondrial activity by 90% after 24 h treatment. Gossypol treatment (100 µg/mL, 24 h) also decreased total soluble protein content to 3% of the control in macrophages. MTT assay is based on the conversion of water-soluble MTT to an insoluble formazan product by viable cells with active metabolism. Assuming more active cells would have more soluble protein, it is expected that gossypol’s decreasing cellular activity corresponds with reducing soluble protein in the cells. This reduction in soluble protein content is not necessarily contradictory to the elevated levels of mRNAs coding for important but minor protein components in the inflammatory response, glucose transport and insulin signaling pathways. These results from both MTT assay and protein determination indicated that gossypol was toxic to mouse RAW264.7 macrophages under high concentration and/or long treatment time. Our results agreed with most of the previous reports [54,55]. Deng at al. (2013) showed that RAW macrophages were almost completely inhibited by 40 µmol/L of gossypol (corresponding to 20.74 µg/mL) for 24 h treatment by MTT assay at 490 nm [54]. Lin et al. (2016) also reported that 80% of RAW macrophages were inhibited by 20 µmol/L of gossypol (corresponding to 10.37 µg/mL) for 5 h treatment by propidium iodide assay measuring Pi incorporation [55]. The toxic effect of gossypol on cell growth is also supported by its ability to inhibit human colon cancer cell viability [42]. It is unknown why Huo et al. (2013) did not observe a similar inhibitory effect of gossypol on RAW macrophages [17].

The novel finding of the current study was that gossypol significantly increased anti-inflammatory TTP family and proinflammatory cytokine gene expression in mouse macrophages. The effect of gossypol stimulation of TTP gene expression was sustained and became much stronger over a longer treatment time. The magnitude of gossypol stimulation of TTP gene expression was increased from approximately 6-fold after 2–8 h treatment to 20-fold after 24 h treatment. Gossypol also increased TTP homologues ZFP36L1, ZFP36L2 and ZFP36L3 mRNA levels 58-, 26- and 69-fold in 24 h treated macrophages, respectively. In addition, gossypol markedly increased the expression of a number of TTP-targeted proinflammatory cytokine mRNAs in mouse RAW264.7 macrophages, including TNF, COX2/PGES2, GM-CSF, INFγ and IL12 up to 39-, 458-, 136-, 103- and 62-fold, respectively.

The patterns of gossypol’s effect on anti- and proinflammatory gene expression were similar, but not identical, to those of the bacterial endotoxin lipopolysaccharides (LPS) and plant polyphenols from green tea leaves and cinnamon bark. It was shown previously that LPS rapidly induced TTP mRNA, but only had minor effects on the expression of the three TTP homologues (ZFP36L1, ZFP36L2 and ZFP36L3) in mouse RAW264.7 macrophages [30,44]. LPS also induced proinflammatory cytokine gene expression, including TNF, COX2 and IL6 in the macrophages [30]. Cinnamon polyphenolic extract also increased the levels of mRNAs coding for both anti-inflammatory TTP and proinflammatory cytokines, including TNF, COX2 and IL6 in mouse RAW264.7 macrophages [30] and 3T3-L1 adipocytes [31]. However, green tea polyphenols increased TTP gene expression but decreased TNF gene expression in rats with metabolic syndrome caused by feeding on a high-fructose diet [27]. The results presented here do not necessary support the earlier proposal that gossypol has anti-inflammatory properties.

It is not uncommon that agents induce both anti- and proinflammatory gene expression at the same time. The anti-inflammatory TTP mRNA is induced by a number of agents, including growth factors [56,57], cytokines (TNFα, GM-CSF and INFγ) [21,25,57,58], zinc [59] and plant nutritional products (cinnamon and green tea) [27,30]. TTP gene expression is also induced by tumor promoters [56,58], bacterial endotoxin LPS [21,44] and viral infection [60]. It was proposed that TTP regulates proinflammatory cytokine mRNA stability through a feedback inhibition mechanism and/or autoregulation [21]. Biochemically, TTP family proteins regulate gene expression at the posttranscriptional level by binding to and destabilizing proinflammatory cytokine mRNA molecules in mammalian cells [21,22]. It was puzzling for a long time in the research field that many agents including gossypol reported here induced anti-inflammatory TTP family mRNA levels but did not decrease proinflammatory cytokine mRNA levels in mammalian cells. One of the causes for the disconnection was probably translation arrest due to elevated TTP family mRNAs targeted to and packed in stress granules under stresses such as gossypol treatment, leading to the death of mouse macrophages [61].

Agents that induce TTP gene expression may have potential therapeutic value for the prevention and/or treatment of inflammation-related diseases. However, the fact that most of these agents also increase the expression levels of proinflammatory cytokines such as TNFα in the same cells and/or tissues [21] may limit the therapeutic potential of these agents. Therefore, it is still important to search for other agents with the potential to favor anti-inflammatory and reduce proinflammatory gene expression.

Another important finding of this study is that gossypol treatment resulted in elevated expression of glucose transporter and insulin signaling pathway genes. Glucose is critically important for host immunity [38,39]. Glucose uptake in mammalian cells is facilitated by GLUT family proteins [51]. These effects of gossypol are similar to those of the other plant polyphenols. For example, green tea polyphenols regulate gene expression in rats under a high-fructose diet [26]. Cinnamon polyphenols regulate the expression of genes coding for glucose transporters and proteins in the insulin signaling pathway [29,30]. Additionally, macrophages express insulin receptors and initiate a cascade of signaling event which are important by either enhancing or attenuating both proinflammatory and anti-inflammatory macrophage responses [40]. These results suggest that gossypol might be able to affect cell immunity by promoting glucose uptake and increasing insulin sensitivity in the immunologically important macrophages, which is in agreement with a previous finding that gossypol has the potential to manage and prevent diabetes by ameliorating glucose uptake and improving glucose homeostasis using a streptozotocin-induced diabetic mouse model [62].

Future work needs to be carried out to confirm gossypol effects on gene expression at the protein levels and post-transcriptional levels such as phosphorylation status as well as the potential mechanism. Unlike insulin, gossypol can penetrate cell membranes and enter the cell, but little is known about the mechanism of its regulation of gene expression at the current time. Comprehensive metabolic analyses would be ideal to yield more direct evidence for gossypol’s role in mediating carbohydrate and lipid metabolism. It is also necessary to determine if the effect of gossypol on gene expression was the cause leading to cell death or caused by cell death in the mouse macrophages. Finally, it is important to confirm these results observed in mouse macrophages with primary macrophages as well as animals before practical uses. However, there is no absolute correlation among cell death, elevated mRNA levels and coded proteins due to there being many regulatory mechanisms from mRNA to phenotype, such as post-transcriptional and translational regulation, mRNA and protein targeting and degradation, as well as being packed into stress granules or inclusion bodies to become inactive. Nevertheless, the results reported here illustrate a potent effect of cottonseed-derived plant polyphenol gossypol in cell growth and gene expression in mouse macrophages.

## 5. Conclusions

This study demonstrated that gossypol induced macrophage death and reduced protein content, which was accompanied with elevated levels of anti-inflammatory TTP family and proinflammatory cytokine gene expression, as well as glucose transporter and insulin signaling pathway gene expression in mouse macrophages. We recently showed that gossypol strongly stimulated DGAT, HuR and VEGF gene expression in mouse macrophages [48,52,53]. Taken together, these studies indicate that gossypol derived from cottonseed may be a powerful stimulator of gene expression involved in inflammatory responses, glucose transport, insulin signaling and lipid biosynthesis in mouse macrophages, regardless of its direct or indirect effects. The results suggest that gossypol may have therapeutical potential for modulating inflammation, glucose transport and insulin signaling-related diseases such as arthritis, diabetes and obesity, although more research is needed to confirm the findings at the mRNA level with protein and metabolite levels as well as using animal models.

## Figures and Tables

**Table 1:** Gossypol induced cell death of mouse macrophages. Mouse macrophages were treated with various concentrations of gossypol for 2 and 24 h. Cellular toxicity was determined with MTT-Based In Vitro Toxicology Assay. The data represent the mean ± standard (*n* = 3). “*” and “**” displayed in the Table represent significant differences between the control and the treatment at *p* < 0.05 and *p* < 0.01, respectively.

Gossypol Concentration (µg/mL)	A570 nm ± SD (2 h) (% of Control)	A570 nm ± SD (24 h) (% of Control)
0	100.0 ± 8.1	100.0 ± 18.5
0.1	110.2 ± 9.0	92.8 ± 3.2
0.5	93.6 ± 4.8	90.4 ± 5.5
1	99.2 ± 1.3	88.3 ± 11.5
5	92.7 ± 4.9	7.6 ± 0.4 **
10	85.7 ± 7.4 *	7.6 ± 0.4 **
50	69.3 ± 2.4 **	8.6 ± 0.5 **
100	20.0 ± 0.6 **	8.5 ± 3.2 **

**Table 2:** Gossypol reduced soluble protein content in mouse macrophages. Mouse macrophages were treated with gossypol (100 µg/mL) for 2, 4, 8 and 24 h. Protein content was determined with the Bradford method. The data represent the mean ± standard deviation (*n* = 3). “*” and “**” displayed in the Table represent significant differences between the control and the treatment at *p* < 0.05 and *p* < 0.01, respectively.

Treatment	Time (h)	Supernatant Protein			Pellet Protein			Total Protein	
		Concentration (µg/µL)	Amount (mg)	Ratio (%)	Concentration (µg/µL)	Amount (mg)	Ratio (%)	Amount (mg)	Ratio (%)
control	2	7.78 ± 0.36	3.89	100	22.05 ± 0.21	1.1	100	4.99	100
gossypol	2	6.56 ± 0.39 *	3.28	84	23.67 ± 0.22 **	1.18	107	4.47	90
gossypol	4	6.45 ± 0.38 *	3.22	83	31.47 ± 0.15 **	1.57	143	4.80	96
gossypol	8	4.85 ± 0.32 **	2.42	62	34.21 ± 0.45 **	1.71	155	4.13	83
gossypol	24	0.21 ± 0.18 **	0.11	3	22.12 ± 0.45	1.11	101	1.22	24

**Table 3:** Basal levels of mRNAs analyzed in mouse macrophages.

mRNA	Cycle of Threshold (C_T_ ± SD)	Cycle of Threshold (C_T_)	*C_T_*_Target_ − *C_tref (ΔCT)_*	Δ*C_T_*_Target_ − Δ*C_T_*_cal (ΔΔCT)_	Fold (Ttp = 1)
Rpl32	17.82 ± 0.81	17.82	0.00		
Ttp/Zfp36/Tis11	24.76 ± 1.12	24.76	6.94	0.00	1.00
Akt1	23.65 ± 1.69	23.65	5.83	−1.11	2.16
App	22.51 ± 0.81	22.51	4.69	−2.25	4.76
Cox2	30.15 ± 2.38	30.15	12.33	5.39	0.02
Glut1/Slc2a1	27.55 ± 1.49	27.55	9.73	2.79	0.14
Glut3/Slc2a3	26.54 ± 0.75	26.54	8.72	1.78	0.29
Glut4/Slc2a4	34.68 ± 1.20	34.68	16.86	9.92	0.001
Gm-csf	27.58 ± 2.06	27.58	9.76	2.82	0.14
Ifnγ	27.70 ± 2.42	27.70	9.88	2.94	0.13
Il12b	28.03 ± 1.89	28.03	10.21	3.27	0.10
Insr	26.39 ± 073	26.39	8.57	1.63	0.32
Lepr	28.33 ± 2.11	28.33	10.51	3.57	0.08
Pik3r1	26.21 ± 0.58	26.21	8.39	1.45	0.37
Tnf	29.19 ± 2.68	29.19	11.37	4.43	0.05
Zfp36l1/Tis11b	26.07 ± 0.35	26.07	8.25	1.31	0.40
Zfp36l2/Tis11d	24.61 ± 1.03	24.61	6.79	−0.15	1.11
Zfp36l3	29.20 ± 1.13	29.20	11.38	4.44	0.05

## Data Availability

The datasets generated during the current study are available in the NIH Gene Expression Omnibus (GEO) Database, accession number GSE203027. (https://www.ncbi.nlm.nih.gov/geo/query/acc.cgi?acc=GSE203027, access date: 1 March 2023).

## Supplementary Materials

The following supporting information can be downloaded at: https://www.mdpi.com/article/10.3390/biom13040624/s1, Table S1: Sequence Information of qPCR Primers.

## References

[1] Yang C.S., Landau J.M., Huang M.-T., Newmark H.L. (2001). Inhibition of carcinogenesis by dietary polyphenolic compounds. Annu. Rev. Nutr..

[2] Dixon R.A., Xie D.-Y., Sharma S.B. (2005). Proanthocyanidins—A final frontier in flavonoid research?. New Phytol..

[3] Prior R.L., Gu L. (2005). Occurrence and biological significance of proanthocyanidins in the American diet. Phytochemistry.

[4] Hazafa A., Rehman K.-U., Jahan N., Jabeen Z. (2020). The Role of Polyphenol (Flavonoids) Compounds in the Treatment of Cancer Cells. Nutr. Cancer.

[5] Mileo A.M., Nisticò P., Miccadei S. (2019). Polyphenols: Immunomodulatory and Therapeutic Implication in Colorectal Cancer. Front. Immunol..

[6] Long J., Guan P., Hu X., Yang L., He L., Lin Q., Luo F., Li J., He X., Du Z. (2021). Natural Polyphenols as Targeted Modulators in Colon Cancer: Molecular Mechanisms and Applications. Front. Immunol..

[7] Kenar J.A. (2006). Reaction chemistry of gossypol and its derivatives. J. Am. Oil Chem. Soc..

[8] Coutinho E.M. (2002). Gossypol: A contraceptive for men. Contraception.

[9] He Z., Zhang D., Cao H. (2018). Protein profiling of water and alkali soluble cottonseed protein isolates. Sci. Rep..

[10] He Z., Zhang H., Olk D.C. (2015). Chemical Composition of Defatted Cottonseed and Soy Meal Products. PLoS ONE.

[11] Zhong S., Leong J., Ye W., Xu P., Lin S.-H., Liu J.-Y., Lin Y.C. (2013). (−)-Gossypol-enriched cottonseed oil inhibits proliferation and adipogenesis of human breast pre-adipocytes. Anticancer. Res..

[12] Chien C.-C., Ko C.-H., Shen S.-C., Yang L.-Y., Chen Y.-C. (2012). The role of COX-2/PGE2 in gossypol-induced apoptosis of colorectal carcinoma cells. J. Cell. Physiol..

[13] Yuan Y., Tang A.J., Castoreno A.B., Kuo S.-Y., Wang Q., Kuballa P., Xavier R., Shamji A.F., Schreiber S.L., Wagner B.K. (2013). Gossypol and an HMT G9a inhibitor act in synergy to induce cell death in pancreatic cancer cells. Cell Death Dis..

[14] Thakur A., Lum L.G., Schalk D., Azmi A., Banerjee S., Sarkar F.H., Mohommad R. (2012). Pan-Bcl-2 Inhibitor AT-101 Enhances Tumor Cell Killing by EGFR Targeted T Cells. PLoS ONE.

[15] Pang X., Wu Y., Wu Y., Lu B., Chen J., Wang J., Yi Z., Qu W., Liu M. (2011). (−)-Gossypol Suppresses the Growth of Human Prostate Cancer Xenografts via Modulating VEGF Signaling–Mediated Angiogenesis. Mol. Cancer Ther..

[16] Huang Y.-W., Wang L.-S., Dowd M.K., Wan P.J., Lin Y.C. (2009). (−)-Gossypol reduces invasiveness in metastatic prostate cancer cells. Anticancer. Res..

[17] Huo M., Gao R., Jiang L., Cui X., Duan L., Deng X., Guan S., Wei J., Soromou L.W., Feng H. (2013). Suppression of LPS-induced inflammatory responses by gossypol in RAW 264.7 cells and mouse models. Int. Immunopharmacol..

[18] Oskoueian E., Abdullah N., Hendra R., Karimi E. (2011). Bioactive Compounds, Antioxidant, Xanthine Oxidase Inhibitory, Tyrosinase Inhibitory and Anti-Inflammatory Activities of Selected Agro-Industrial By-products. Int. J. Mol. Sci..

[19] Fu M., Blackshear P.J. (2017). RNA-binding proteins in immune regulation: A focus on CCCH zinc finger proteins. Nat. Rev. Immunol..

[20] Patial S., Blackshear P.J. (2016). Tristetraprolin as a Therapeutic Target in Inflammatory Disease. Trends Pharmacol. Sci..

[21] Carballo E., Lai W.S., Blackshear P.J. (1998). Feedback Inhibition of Macrophage Tumor Necrosis Factor-α Production by Tristetraprolin. Science.

[22] Lai W.S., Carballo E., Thorn J.M., Kennington E.A., Blackshear P.J. (2000). Interactions of CCCH zinc finger proteins with mRNA. Binding of tristetraprolin-related zinc finger proteins to Au-rich elements and destabilization of mRNA. J. Biol. Chem..

[23] Phillips K., Kedersha N., Shen L., Blackshear P.J., Anderson P. (2004). Arthritis suppressor genes TIA-1 and TTP dampen the expression of tumor necrosis factor alpha, cyclooxygenase 2, and inflammatory arthritis. Proc. Natl. Acad. Sci. USA.

[24] Taylor G.A., Carballo E., Lee D.M., Lai W.S., Thompson M.J., Patel D.D., I Schenkman D., Gilkeson G.S., E Broxmeyer H., Haynes B.F. (1996). A Pathogenetic Role for TNFα in the Syndrome of Cachexia, Arthritis, and Autoimmunity Resulting from Tristetraprolin (TTP) Deficiency. Immunity.

[25] Sauer I., Schaljo B., Vogl C., Gattermeier I., Kolbe T., Müller M., Blackshear P.J., Kovarik P. (2006). Interferons limit inflammatory responses by induction of tristetraprolin. Blood.

[26] Cao H., Hininger-Favier I., Kelly M.A., Benaraba R., Dawson H.D., Coves S., Roussel A.M., Anderson R.A. (2007). Green Tea Polyphenol Extract Regulates the Expression of Genes Involved in Glucose Uptake and Insulin Signaling in Rats Fed a High Fructose Diet. J. Agric. Food Chem..

[27] Cao H., Kelly M., Kari F., Dawson H.D., Urban J.F., Coves S., Roussel A., Anderson R. (2007). Green tea increases anti-inflammatory tristetraprolin and decreases pro-inflammatory tumor necrosis factor mRNA levels in rats. J. Inflamm..

[28] Cao H., Polansky M.M., Anderson R.A. (2007). Cinnamon extract and polyphenols affect the expression of tristetraprolin, insulin receptor, and glucose transporter 4 in mouse 3T3-L1 adipocytes. Arch. Biochem. Biophys..

[29] Cao H., Graves D.J., Anderson R.A. (2010). Cinnamon extract regulates glucose transporter and insulin-signaling gene expression in mouse adipocytes. Phytomedicine.

[30] Cao H., Urban J.F., Anderson R.A. (2008). Cinnamon Polyphenol Extract Affects Immune Responses by Regulating Anti- and Proinflammatory and Glucose Transporter Gene Expression in Mouse Macrophages. J. Nutr..

[31] Cao H., Anderson R.A. (2011). Cinnamon Polyphenol Extract Regulates Tristetraprolin and Related Gene Expression in Mouse Adipocytes. J. Agric. Food Chem..

[32] Blackshear P.J. (2002). Tristetraprolin and other CCCH tandem zinc-finger proteins in the regulation of mRNA turnover. Biochem. Soc. Trans..

[33] Blackshear P.J., Phillips R.S., Ghosh S., Ramos S.V., Richfield E.K., Lai W.S. (2005). Zfp36l3, a Rodent X Chromosome Gene Encoding a Placenta-Specific Member of the Tristetraprolin Family of CCCH Tandem Zinc Finger Proteins. Biol. Reprod..

[34] Cha H.J., Lee H.H., Chae S.W., Cho W.J., Kim Y.M., Choi H.-J., Choi D.H., Jung S.W., Min Y.J., Lee B.J. (2011). Tristetraprolin downregulates the expression of both VEGF and COX-2 in human colon cancer. Hepato-Gastroenterology.

[35] Carballo E., Lai W.S., Blackshear P. (2000). Evidence that tristetraprolin is a physiological regulator of granulocyte-macrophage colony-stimulating factor messenger RNA deadenylation and stability. Blood.

[36] Kontoyiannis D., Boulougouris G., Manoloukos M., Armaka M., Apostolaki M., Pizarro T., Kotlyarov A., Forster I., Flavell R., Gaestel M. (2002). Genetic dissection of the cellular pathways and signaling mechanisms in modeled tumor necrosis factor-induced Crohn’s-like inflammatory bowel disease. J. Exp. Med..

[37] Molle C., Zhang T., De Lendonck L.Y., Gueydan C., Andrianne M., Sherer F., Van Simaeys G., Blackshear P., Leo O., Goriely S. (2013). Tristetraprolin regulation of interleukin 23 mRNA stability prevents a spontaneous inflammatory disease. J. Exp. Med..

[38] Gamelli R.L., Liu H., He L.-K., Hofmann C.A. (1996). Augmentations of glucose uptake and glucose transporter-1 in macrophages following thermal injury and sepsis in mice. J. Leukoc. Biol..

[39] Sala-Vila A., Barbosa V.M., Calder P. (2007). Olive oil in parenteral nutrition. Curr. Opin. Clin. Nutr. Metab. Care.

[40] Püschel G.P., Klauder J., Henkel J. (2022). Macrophages, Low-Grade Inflammation, Insulin Resistance and Hyperinsulinemia: A Mutual Ambiguous Relationship in the Development of Metabolic Diseases. J. Clin. Med..

[41] Cao H., Sethumadhavan K., Bland J.M. (2018). Isolation of Cottonseed Extracts That Affect Human Cancer Cell Growth. Sci. Rep..

[42] Cao H., Sethumadhavan K., Cao F., Wang T.T.Y. (2021). Gossypol decreased cell viability and down-regulated the expression of a number of genes in human colon cancer cells. Sci. Rep..

[43] Cao H., Sethumadhavan K. (2022). Identification of *Bcl2* as a Stably Expressed qPCR Reference Gene for Human Colon Cancer Cells Treated with Cottonseed-Derived Gossypol and Bioactive Extracts and Bacteria-Derived Lipopolysaccharides. Molecules.

[44] Cao H., Tuttle J.S., Blackshear P.J. (2004). Immunological Characterization of Tristetraprolin as a Low Abundance, Inducible, Stable Cytosolic Protein. J. Biol. Chem..

[45] Cao H. (2004). Expression, Purification, and Biochemical Characterization of the Antiinflammatory Tristetraprolin: A Zinc-Dependent mRNA Binding Protein Affected by Posttranslational Modifications. Biochemistry.

[46] Bustin S.A., Benes V., Garson J.A., Hellemans J., Huggett J., Kubista M., Mueller R., Nolan T., Pfaffl M.W., Shipley G.L. (2009). The MIQE Guidelines: Minimum Information for Publication of Quantitative Real-Time PCR Experiments. Clin. Chem..

[47] Cao H., Shockey J.M. (2012). Comparison of TaqMan and SYBR Green qPCR Methods for Quantitative Gene Expression in Tung Tree Tissues. J. Agric. Food Chem..

[48] Cao H., Sethumadhavan K. (2018). Cottonseed Extracts and Gossypol Regulate Diacylglycerol Acyltransferase Gene Expression in Mouse Macrophages. J. Agric. Food Chem..

[49] Livak K.J., Schmittgen T.D. (2001). Analysis of relative gene expression data using real-time quantitative PCR and the 2^−ΔΔCT^ Method. Methods.

[50] Cao H., Cao F., Roussel A.-M., Anderson R.A. (2013). Quantitative PCR for glucose transporter and tristetraprolin family gene expression in cultured mouse adipocytes and macrophages. In Vitr. Cell. Dev. Biol.-Anim..

[51] Fukuzumi M., Shinomiya H., Shimizu Y., Ohishi K., Utsumi S. (1996). Endotoxin-induced enhancement of glucose influx into murine peritoneal macrophages via GLUT. Infect. Immun..

[52] Cao H., Sethumadhavan K. (2019). Gossypol but not cottonseed extracts or lipopolysaccharides stimulates HuR gene expression in mouse cells. J. Funct. Foods.

[53] Cao H., Sethumadhavan K., Wu X., Zeng X. (2021). Cottonseed-derived gossypol and ethanol extracts differentially regulate cell viability and VEGF gene expression in mouse macrophages. Sci. Rep..

[54] Deng S., Yuan H., Yi J., Lu Y., Wei Q., Guo C., Wu J., Yuan L., He Z. (2013). Gossypol acetic acid induces apoptosis in RAW264.7 cells via a caspase-dependent mitochondrial signaling pathway. J. Vet. Sci..

[55] Lin Q.-R., Li C.-G., Zha Q.-B., Xu L.-H., Pan H., Zhao G.-X., Ouyang D.-Y., He X.-H. (2016). Gossypol induces pyroptosis in mouse macrophages via a non-canonical inflammasome pathway. Toxicol. Appl. Pharmacol..

[56] Lai W.S., Stumpo D.J., Blackshear P.J. (1990). Rapid insulin-stimulated accumulation of an mRNA encoding a proline-rich protein. J. Biol. Chem..

[57] DuBois R., McLane M., Ryder K., Lau L., Nathans D. (1990). A growth factor-inducible nuclear protein with a novel cysteine/histidine repetitive sequence. J. Biol. Chem..

[58] Varnum B.C., Lim R.W., Kujubu D.A., Luner S.J., Kaufman S.E., Greenberger J.S., Gasson J.C., Herschman H.R. (1989). Granulocyte-macrophage colony-stimulating factor and tetradecanoyl phorbol acetate induce a distinct, restricted subset of primary- response TIS genes in both proliferating and terminally differentiated myeloid cells. Mol. Cell. Biol..

[59] Cousins R.J., Blanchard R.K., Popp M.P., Liu L., Cao J., Moore J.B., Green C.L. (2003). A global view of the selectivity of zinc deprivation and excess on genes expressed in human THP-1 mononuclear cells. Proc. Natl. Acad. Sci. USA.

[60] Taddeo B., Zhang W., Roizman B. (2006). The U_L_41 protein of herpes simplex virus 1 degrades RNA by endonucleolytic cleavage in absence of other cellular or viral proteins. Proc. Natl. Acad. Sci. USA.

[61] Stoecklin G., Stubbs T., Kedersha N., Wax S., Rigby W.F., Blackwell T.K., Anderson P. (2004). MK2-induced tristetraprolin:14-3-3 complexes prevent stress granule association and ARE-mRNA decay. EMBO J..

[62] Alam B., An H., Ra J.-S., Lim J.-Y., Lee S.-H., Yoo C.-Y., Lee S.-H. (2018). Gossypol from Cottonseeds Ameliorates Glucose Uptake by Mimicking Insulin Signaling and Improves Glucose Homeostasis in Mice with Streptozotocin-Induced Diabetes. Oxid. Med. Cell. Longev..

