# Supplementary material for: Plant Polyphenol Gossypol Induced Cell Death and Its Association with Gene Expression in Mouse Macrophages
Source: Biomolecules. 2023 Mar 30;13(4):624. doi: 10.3390/biom13040624 (PMC10136236; doi:10.3390/biom13040624)
Supplement: Supplementary file 1 [file biomolecules-13-00624-s001.zip › biomolecules-2235351-supplementary.pdf]

Supplementary

Table S1. Sequence Information of qPCR Primers.

mRNA	accession no.	amplicon (bp)	forward primer (5′ to 3′)	reverse primer (5′ to 3′)
**TTP family**				
Ttp/Zfp36/Tis11	NM_011756	70	GGTACCCCAGGCTGGCTTT	ACCTGTAACCCCAGAACTTGGA
Zfp36l1/Tis11b	NM_007564	60	TGCGAACGCCCACGAT	CTTCGCTCAAGTCAAAAATGG
Zfp36l2/Tis11d	NM_001001806	77	GAGGGCACCTCCCAACCT	TGACAGAAGTGTGGTCGACATTT
Zfp36l3	NM_001009549	70	CGAACTGCGTACCCTGTCAAG	GCCAACGCTGTGGAAGGT
**Cytokines**				
Gm-csf/Csf2	NM_009969	71	CACCCGCTCACCCATCAC	GGAGGTTCAGGGCTTCTTTGA
Cox2/Ptgs2	NM_011198	106	CCACCTCTGCGATGCTCTTC	CATTCCCCACGGTTTTGACATG
Ifnγ	NM_008337	81	TGGCATAGATGTGGAAGAAAAGAG	TGCAGGATTTTCATGTCACCAT
Il12b	NM_008352	79	GACCAGAGACATGGAGTCATAGG	TGTACTGGCCAGCATCTAGAAACT
Tnf/Tnfα	NM_013693	74	GCTGTCGCTACATCACTGAACCT	TGACCCGTAGGGCAATTACA
**GLUT family**				
Glut1 (Slc2a1)	M13979	123	CGTGCTTATGGGTTTCTCCAAA	GACACCTCCCCCACATACATG
Glut2 (Slc2a2)	NM_012879	80	TTTGCAGTAGGCGGAATGG	GCCAACATGGCTTTGATCCTT
Glut3 (Slc2a3)	NM_017102	112	TGAAGCCATGAGCTTTGTCTGT	GCCCTGGCTGAAGAGTTCAG
Glut4 (Slc2a4)	NM_012751	87	CAACTGGACCTGTAACTTCATCGT	ACGGCAAATAGAAGGAAGACGTA
**Insulin signaling**				
Insr	NM_017071	137	CAAAAGCACAATCAGAGTGAGTATGAC	ACCACGTTGTGCAGGTAATCC
Akt1/Pkb	NM_033230	90	TGGACTACTTGCACTCCGAGAA	TTATCTTGATATGCCCGTCCTT
GSK3β	NM_032080	106	TTAAGGAAGGAAAAGGTGAATCGA	CCAAAAGCTGAAGGCTGCTG
Pik3r1	NM_013005	118	CCTCTCCTTATAAAGCTCCTGGAA	GATCACAATCAAGAAGCTGTCGTAA
**Other mRNA**				
App	NM_007471	70	GTTGCCTAGTTGGTGAGTTTGT	TCCTGGTGTAGGAACTTGCACTTG
Lepr	NM_146146	92	TGACCAGTGTAACAGTGCTAACTTCTC	CATATTTAACTGAGGGTTGTCTCTGACA
Rpl32 (reference)	NM_172086	66	AACCGAAAAGCCATTGTAGAAA	CCTGGCGTTGGGATTGG
